# Developing a clinical case definition for Chikungunya fever in the Democratic Republic of the Congo

**DOI:** 10.1371/journal.pone.0355216

**Published:** 2026-08-06

**Authors:** Florian Vogt, Antoine Nkuba-Ndaye, Anja De Weggheleire, Sheila Makiala-Mandanda, Placide Mbala-Kingebeni, Benoit Mputu-Ngoyi, Eddy Kinganda-Lusamaki, Elisabeth Pukuta, Lorenzo Subissi, Birgit De Smet, Jozefien Buyze, Steve Ahuka-Mundeke

**Affiliations:** 1 The Kirby Institute, University of New South Wales, Sydney, Australia; 2 National Centre for Epidemiology and Population Health, Australian National University, Canberra, Australia; 3 Institute of Tropical Medicine Antwerp, Antwerp, Belgium; 4 Recherches Translationnelles sur le Virus de l’Immunodéficience Humaine et les Maladies Infectieuses, Université de Montpellier, Institut de Recherche pour le Développement, INSERM, Montpellier, France; 5 Institut National de Recherche Biomédicale, Kinshasa, Democratic Republic of the Congo; 6 Département de Biologie Médicale, Cliniques Universitaires de Kinshasa, Kinshasa, Democratic Republic of the Congo; 7 Sciensano, Brussels, Belgium; CEA, FRANCE

## Abstract

**Introduction:**

Chikungunya fever presents a diagnostic challenge in Sub-Saharan Africa due to the absence of a region-specific case definition, especially in settings with high disease burden and diverse aetiologies of acute febrile illnesses. Our study aimed to develop an evidence-based clinical case definition for acute Chikungunya virus (CHIKV) infection suitable for use at resource-limited primary health care level.

**Methods:**

We conducted a cross-sectional study in Kinshasa, Democratic Republic of the Congo, between June and November 2019 during an ongoing CHIKV outbreak. Patients aged ≥3 years with symptoms suggestive of acute CHIKV infection were enrolled. Clinical examination data and blood samples were collected for CHIKV diagnosis via PCR and ELISA. Malaria rapid diagnostic tests were also performed. We used Classification and Regression Tree (CART) analysis with 10-fold cross-validation and predictive logistic regression models with lasso penalty, with performance assessed via the Area under the Receiver Operating Characteristics Curve (AUC).

**Results:**

Of 132 analysed patients, 40.2% had acute CHIKV infection. CART analysis identified knee and shoulder pain as potential predictors, however classifying only 67.4% of patients correctly while missing 71.7% of cases (cross-validated AUC 53%; 95%CI 43%−63%). Logistic regression achieved 61.1% accuracy, with a corresponding cross-validated AUC of 62% (95%CI 52%−72%). Exploratory analysis excluding malaria co-infections yielded similar CART performance, while logistic regression failed to identify any reliable predictors.

**Conclusions:**

While our study provides valuable insights into the clinical presentation of CHIKV in Sub-Saharan Africa, it highlights the challenges of developing a simple symptom-based diagnostic algorithm suitable for resource-limited primary health care settings. Certain joint pain patterns showed predictive value, however no model achieved sufficient accuracy for clinical use. Improved access to serological testing and integration of epidemiological context are essential for CHIKV diagnosis in such contexts. Further research is needed to refine clinical criteria and support outbreak response in resource-limited regions.

## Introduction

Chikungunya fever is a mosquito-borne viral disease caused by the Chikungunya virus (CHIKV), an enveloped positive single-strain RNA alphavirus of the *Togaviridae* family. [1] Three genotypes have been described to date: Asian, West African and East/Central/South African (ECSA). [2] In Africa, CHIKV transmission is maintained in a sylvatic cycle involving wild non-human primates and various arboreal *Aedes* mosquitoes. Mosquito-to-human transmission in urban transmission cycles is dominated by *A. aegypti* and *A. albopictus*. [1,3] Since the first recorded epidemic in Tanzania in 1952, CHIKV outbreaks have been reported in many countries around the world and with increasing frequency in Sub-Saharan Africa during recent years.

The proportion of symptomatic CHIKV infections during epidemics has been reported to be between 70–97%. [2] For people who develop symptomatic illness, the incubation period is usually between three to seven days (range one to twelve days). Patients typically present with abrupt onset of fever, and severe (mostly symmetric) joint pain followed in some cases by a rash. Other symptoms may include headache, conjunctival redness, nausea and vomiting. Severe manifestations are rare, but can include myocarditis, hepatitis, ocular and neurological disorders. [1,2] The viraemic period of CHIKV lasts two to ten days, which matches the acute symptomatic phase of the disease and possible detection through viral nucleic acid-based tests. Laboratory confirmation of CHIKV infection is done using CHIKV-specific real-time reverse-transcription polymerase chain reaction (RT-PCR) tests, Enzyme linked Immunosorbent Assays (ELISA), and CHIKV-specific IgM by immunofluorescence assays (IFA). [4,5] PCR technology and serological methods are complex, expensive, and only available at few central laboratories in most low-resource settings.

A good clinical case definition for use at primary health care level is crucial to ensure timely and appropriate care, to avoid that laboratory capacity is overburdened if too many laboratory confirmations are requested by health workers during outbreaks, that the number of cases is not severely under- or overestimated during surveillance and outbreak response, or that entire outbreaks are missed. A sound balance of sufficiently high specificity and sensitivity is therefore key. However, establishing an accurate and practical case definition for acute CHIKV infection applicable to different contexts and populations is difficult due to the wide range of clinical symptoms and atypical presentations, possible overlap with other pathologies causing an acute febrile illness syndrome, differences in clinical presentations across CHIKV strains, and differences in host populations. [6]

A couple of case definitions have been developed for the Americas [7,8] and applied to South-East Asia [9], featuring arthritis, fatigue, rash and ankle joint pain [7], and fever >38.5 °C and acute onset joint pain [8], respectively. However, no evidence-based case definition exists for Sub-Saharan Africa to date. In a context where many other severe, potentially fatal infectious diseases such as malaria, typhoid fever, and dengue co-circulate in the same population, this absence leaves clinicians with little guidance how to correctly identify, differentiate and manage patients on a clinical basis.

We conducted an exploratory clinical study during a CHIKV outbreak in Kinshasa, the Democratic Republic of the Congo (DRC) in 2019 with the aim to identify symptom patterns to develop a clinical case definition for suspicion of chikungunya fever for use at primary health facility level.

## Methods

### Study design

This was a cross-sectional study embedded into routine health services with prospectively-planned collection of blood samples and clinical information from patients seeking health care at the outpatient department of the health centre ‘Centre Hospitalier Lukunga’ (CH Lukunga), Binza Ozone health zone in Kinshasa, DRC between June and November 2019. Being an exploratory study, no prior null hypotheses or formal sample size estimations was done.

### Context

In November 2018, local clinicians in the health zones of Mont-Ngafula I and Mont-Ngafula II, located in the province of Kinshasa, noted an increase in acute febrile cases associated with severe joint pain and headache. In January 2019, the Provincial Division of Health (PDH) and the National Institute of Biomedical Research (INRB) conducted a joint epidemic outbreak investigation and confirmed CHIKV infection in blood of suspect patients through RT-PCR. [10] Following further alerts, additional investigations in Matadi, Kongo Central province in collaboration with the Institute of Tropical Medicine Antwerp (ITM), showed that the outbreak in Western Kinshasa had spread further into Kongo central province. [11,12] Chikungunya fever is not part of the national surveillance system in DRC, and no official case definition exists for the DRC to date. [13] Laboratory confirmation of acute CHIKV infection in the DRC is limited to the Virology department of INRB.

### Study setting

CH Lukunga was selected as study site following consultations of provincial health authorities, the INRB laboratory database to identify the health zones with the most active CHIKV alerts, and subsequent exchanges with the health management teams of the health zones of Binza Ozone and Binza Meteo. CH Lukunga is located in the municipality Ngaliema in the western part of Kinshasa and offers general preventive and curative health services at primary care level through outpatient consultations for adults and children, as well as a limited inpatient services in internal medicine, surgery, and maternity care. Staff consists of midwives, nurses, laboratory technicians, and two general physicians. CH Lukunga is a private not-for-profit entity owned and managed by the Diocesan Office of Medical Works (BDOM), but functions and reports according to Ministry of Health guidelines. It has basic laboratory infrastructure for blood sampling, blood sample storage, rapid diagnostic tests, blood grouping, and microscopy.

### Clinical procedures

Enrolment started 25 June and continued until 28 November 2019. Eligible patients were invited consecutively to participate in the study by a trained nurse or medical doctor of the CH Lukunga during their outpatient consultation. Inclusion criteria were: being aged 3 years or above; consulting for symptoms suggestive of acute CHIKV infection (self-reported sudden-onset of fever or arthralgia, currently ongoing or during the past 7 days); being willing and able to provide written informed consent (or by a guardian for minors). Patients who were considered unsuitable for venous blood sample collection as per clinical judgement of the recruiting health care worker (e.g., very sick or fragile patients) were excluded. Standard operating procedures were developed for all study-related activities prior to start of enrolment. During medical consultations, socio-demographic characteristics, detailed clinical signs and symptoms, and previous self-medication were recorded using a paper-based case report form (see S1 Annex). Finger prick blood was taken for on-site malaria rapid diagnostic testing (RDT), plus 5 ml venous blood were drawn into EDTA tubes for CHIKV diagnostic testing at INRB. Routine patient care continued to be offered according to the usual standards and guidance applied in CH Lukunga and was not altered by the study. The malaria RDT result was immediately shared with the treating physician or nurse.

### Laboratory procedures

EDTA tubes were stored at 4–8 °C at the study site and transported to INRB at least once a week in a cooled transportation box. At INRB, blood samples were aliquoted and stored at −20 °C. All samples were tested in parallel for CHIKV RNA presence using RT-PCR (RNA extraction with the QIAamp Viral RNA Mini Kit (Qiagen, Germantown, MD, USA) and a CHIKV specific RT-qPCR from Bio-Rad Laboratories, Marnes-La-Coquette, France, as described previously [11], and for IgM and IgG CHIKV antibodies using the enzyme-linked immunosorbent assay from Euroimmun, Lübeck, Germany. Active CHIKV infection was defined as having a PCR cycle threshold value below 35 or showing presence of IgM antibodies (defined as optical density ratio ≥1.1). For both IgM and IgG, optical density ratios ≥0.8 and <1.1 were considered borderline, and negative if <0.8). Malaria testing was done with the SD Bioline Malaria Ag P.f/Pan (HRP2/pLDH) antigen rapid diagnostic test.

### Data analysis

Data were double-entered into an *EpiData v.3.1* database and analyzed using the software packages *Stata v.14* and *R v.3.6.1*. From the clinical information collected through the case report form, we created the following composite variables: pain or swelling in small/distant joints upper limbs (wrist or hand); pain or swelling in small/distant joints lower limbs (ankle, foot); swelling of any joint; pain in any joint; pain in >3 joints; swelling in >3 joints; skin rash or pruritus.

Most clinical and demographic data were binary or converted into categorical variables, and hence presented using percentages for descriptive analysis by acute CHIKV infection status. Borderline antibody titres were considered negative in the analysis. To identify the combination of clinical symptoms that best predict acute CHIKV infection, we used classification and regression tree (CART) analysis where the tree was pruned with cost-complexity parameter chosen by cross-validation, and logistic regression with lasso penalty chosen by 10-fold cross-validation. The performance of the prediction model was assessed by estimating the area under the Receiver Operating Characteristics (ROC) curve (AUC). Since the same data were used to build the tree and for prediction, a 10-fold cross-validation of the AUC was also done, to correct for potential over-optimization of the naïve AUC. Given the high prevalence of malaria infection in the study setting, we also conducted an exploratory secondary analysis to establish a diagnostic definition for acute CHIKV infection as differential diagnosis to malaria (i.e., after malaria is ruled out). For this we excluded all current and recent malaria infections (defined by malaria RDT pf + pan positive test result) from the analysis.

### Ethics

This was an observational study that did not interfere with routine care. Patients received standard of care according to routine practice regardless of study participation. Individual written informed consent was obtained from each participant or guardian before any study procedures were started. The study was approved by the Institutional Review Board of ITM, Antwerp, Belgium (ref #1312/19), and the Ethics Review Board of Kinshasa University, Kinshasa, DRC. Additional information regarding the ethical, cultural, and scientific considerations specific to inclusivity in global research is included in the Supporting Information (S4 Checklist).

## Results

We enrolled 134 patients into our study, of which two (1.5%) were excluded from the analysis due to missing outcome data. Among the remaining 132 patients, 86 (65.2%) were female and 34 (25.8%) were below 10 years old. One hundred and nineteen (93.2%) had an axillary temperature ≥37.5°C, 115 (87.1%) had a headache, 105 (79.5%) had arthralgia, 72 (54.5%) had myalgia, and 25 (18.9%) had either skin rash or pruritus (see Table 1 and S2 Table for further demographic and clinical characteristics). Fifty-three (40.2%) were confirmed as having acute CHIKV infection as per laboratory testing. Of those, 10 (18.9%) were positive only on RT-PCR, 8 (15.1%) on both RT-PCR and IgM, 2 (3.8%) on RT-PCR and borderline on IgM, and 33 (62.3%) only on IgM. IgG positivity, indicative of past infection, was 47.0% (n = 62) among the total study population, and 58.5% (n = 31) among those with acute CHIJKV infection. Thirty-four (25.8%) patients had a pf + pan positive malaria RDT result, indicating current or recent malaria infection, while 10 (7.6%) patients had a malaria-CHIKV co-infection (Table 2).

**Table 1:** Demographic and clinical characteristics by CHIKV infection status among all included participants (N = 132).

	CHIKV Infection					
	Negative		Positive		Total	
	N = 79	%	N = 53	%	N = 132	%
**Patient Age** (Years)						
<10	22	27.8	12	22.6	34	25.8
≥10 < 20	14	17.7	16	30.2	30	22.7
≥20, < 30	15	19.0	8	15.1	23	17.4
≥30 < 40	9	11.4	4	7.5	13	9.8
≥40, < 50	7	8.9	6	11.3	13	9.8
≥50	12	15.2	7	13.2	19	14.4
**Patient Sex**						
Female	50	63.3	36	67.9	86	65.2
Male	29	36.7	17	32.1	46	34.8
**Fever** (°C)						
<37.5	6	7.6	3	5.7	9	6.8
≥37.5, < 38	32	40.5	18	34.0	50	37.9
≥38, < 38.5	27	34.2	16	30.2	43	32.6
≥38.5, < 39	6	7.6	9	17.0	15	11.4
≥39	7	8.9	4	7.5	11	8.3
Missing	1	1.3	3	5.7	4	3.0
**Past Symptoms**						
No	59	74.7	45	84.9	104	78.8
Yes	19	24.1	8	15.1	27	20.5
Missing	1	1.3	0	0.0	1	0.8
**Time Since Symptom Onset** (Days)						
≤2	20	25.3	15	28.3	35	26.5
3	23	29.1	14	26.4	37	28.0
4	16	20.3	12	22.6	28	21.2
>4	20	25.3	11	20.8	31	23.5
Missing	0	0.0	1	1.9	1	0.8
**Chills**						
No	9	11.4	4	7.5	13	9.8
Yes	70	88.6	49	92.5	119	90.2
**Headache**						
No	12	15.2	5	9.4	17	12.9
Yes	67	84.8	48	90.6	115	87.1
**Vertigo**						
No	43	54.4	27	50.9	70	53.0
Yes	36	45.6	26	49.1	62	47.0
**Cough**						
No	50	63.3	32	60.4	82	62.1
Yes	29	36.7	21	39.6	50	37.9
**Rhinitis**						
No	48	60.8	34	64.2	82	62.1
Yes	31	39.2	19	35.8	50	37.9
**Dyspnea**						
No	53	67.1	37	69.8	90	68.2
Yes	26	32.9	16	30.2	42	31.8
**Chest pain**						
No	58	73.4	33	62.3	91	68.9
Yes	21	26.6	20	37.7	41	31.1
**Skin rash**						
None	67	84.8	44	83.0	111	84.1
Generalized	9	11.4	5	9.4	14	10.6
Partial	3	3.8	4	7.5	7	5.3
**Pruritus**						
No	67	84.8	43	81.1	110	83.3
Yes	12	15.2	10	18.9	22	16.7
**Rash or Pruritus**						
No	66	83.5	41	77.4	107	81.1
Yes	13	16.5	12	22.6	25	18.9
**Anorexia**						
No	25	31.6	16	30.2	41	31.1
Yes	54	68.4	37	69.8	91	68.9
**Asthenia**						
No	8	10.1	9	17.0	17	12.9
Yes	71	89.9	44	83.0	115	87.1
**Abdominal Pain**						
No	36	45.6	34	64.2	70	53.0
Yes	43	54.4	19	35.8	62	47.0
**Nausea and Vomiting**						
No	38	48.1	31	58.5	69	52.3
Yes	41	51.9	22	41.5	63	47.7
**Diarrhea**						
No	67	84.8	46	86.8	113	85.6
Yes	12	15.2	7	13.2	19	14.4
Total	79	100.0	53	100.0	132	100.0
**Bleeding**						
No	79	100.0	52	98.1	131	99.2
Yes	0	0.0	1	1.9	1	0.8
**Retro-Orbital Pain**						
No	62	78.5	38	71.7	100	75.8
Yes	17	21.5	15	28.3	32	24.2
**Conjunctival Injection**						
No	62	78.5	41	77.4	103	78.0
Yes	17	21.5	12	22.6	29	22.0
**Myalgia**						
No	39	49.4	21	39.6	60	45.5
Yes	40	50.6	32	60.4	72	54.5
**Lumbago**						
No	41	51.9	27	50.9	68	51.5
Yes	38	48.1	26	49.1	64	48.5
**Arthralgia**						
No	21	26.6	6	11.3	27	20.5
Yes	58	73.4	47	88.7	105	79.5
**Pain or Swelling in Distal Upper Joints**						
No	39	49.4	20	37.7	59	44.7
Yes	40	50.6	33	62.3	73	55.3
**Pain or Swelling in Distal Lower Joints**						
No	38	48.1	24	45.3	62	47.0
Yes	41	51.9	29	54.7	70	53.0
**Pain in>=1 Joint**						
No	21	26.6	6	11.3	27	20.5
Yes	58	73.4	47	88.7	105	79.5
**Swelling in>=1 Joint**						
No	67	84.8	41	77.4	108	81.8
Yes	12	15.2	12	22.6	24	18.2
**Pain in >3 Joints**						
No	53	67.1	29	54.7	82	62.1
Yes	26	32.9	24	45.3	50	37.9
**Swelling in >3 Joints**						
No	79	100.0	53	100.0	132	100.0
Yes	0	0.0	0	0.0	0	0.0

CHIKV: Chikungunya virus. °C: Degrees Celsius. N: Column total. %: Percentage.

**Table 2:** Malaria-CHIKV co-infection status among all included participants (N = 132).

	CHIKV Infection					
	Negative		Positive		Total	
	N = 79	%	N = 53	%	N = 132	%
**Malaria RDT Result**						
PF	17	21.5	7	13.2	24	18.2
PF + PAN	24	30.4	10	18.9	34	25.8
Negative	38	48.1	36	67.9	74	56.1

CHIKV: Chikungunya virus. PF: lasmodium falciparum. PAN: Pan-malarial antigen. N: Column total. %: Percentage.

**Fig 1:**
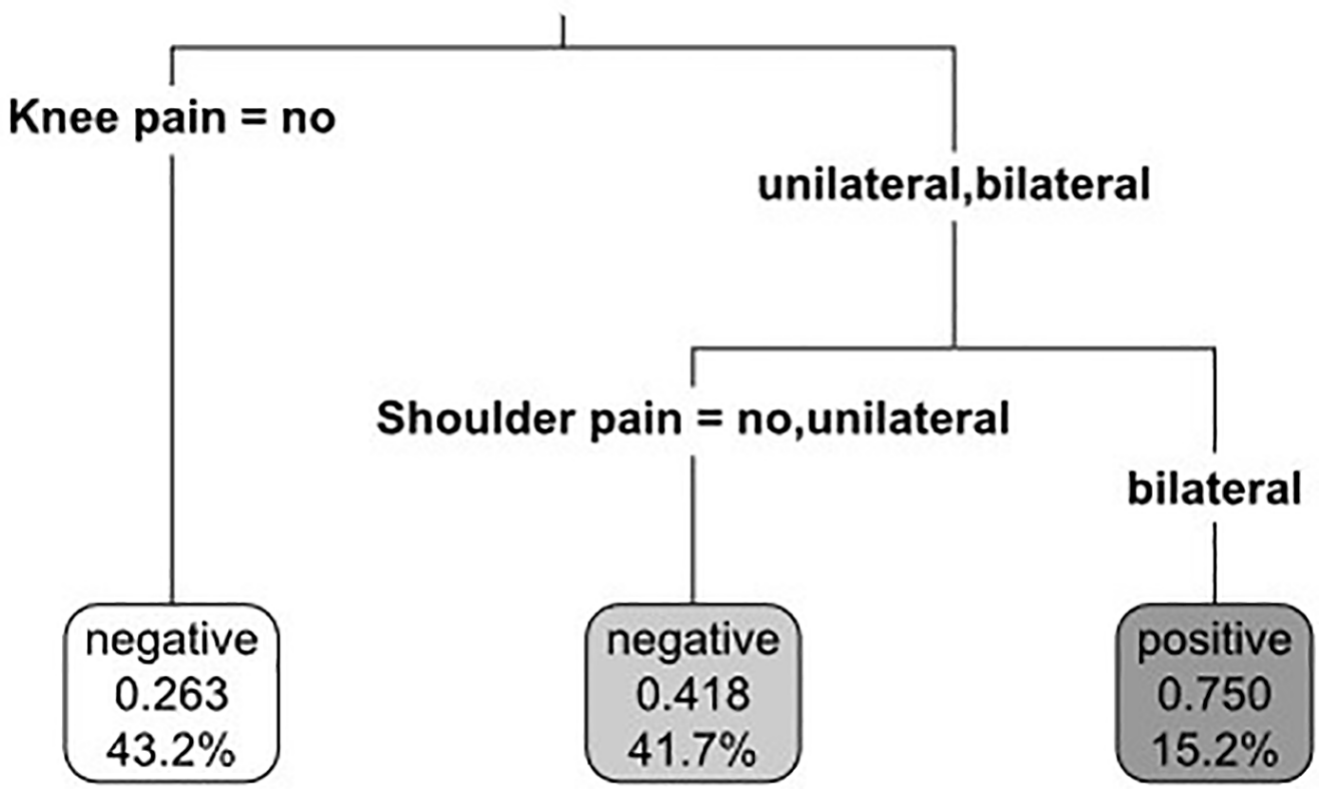
Classification and regression tree analysis among all included participants (N = 132).

**Fig 2:**
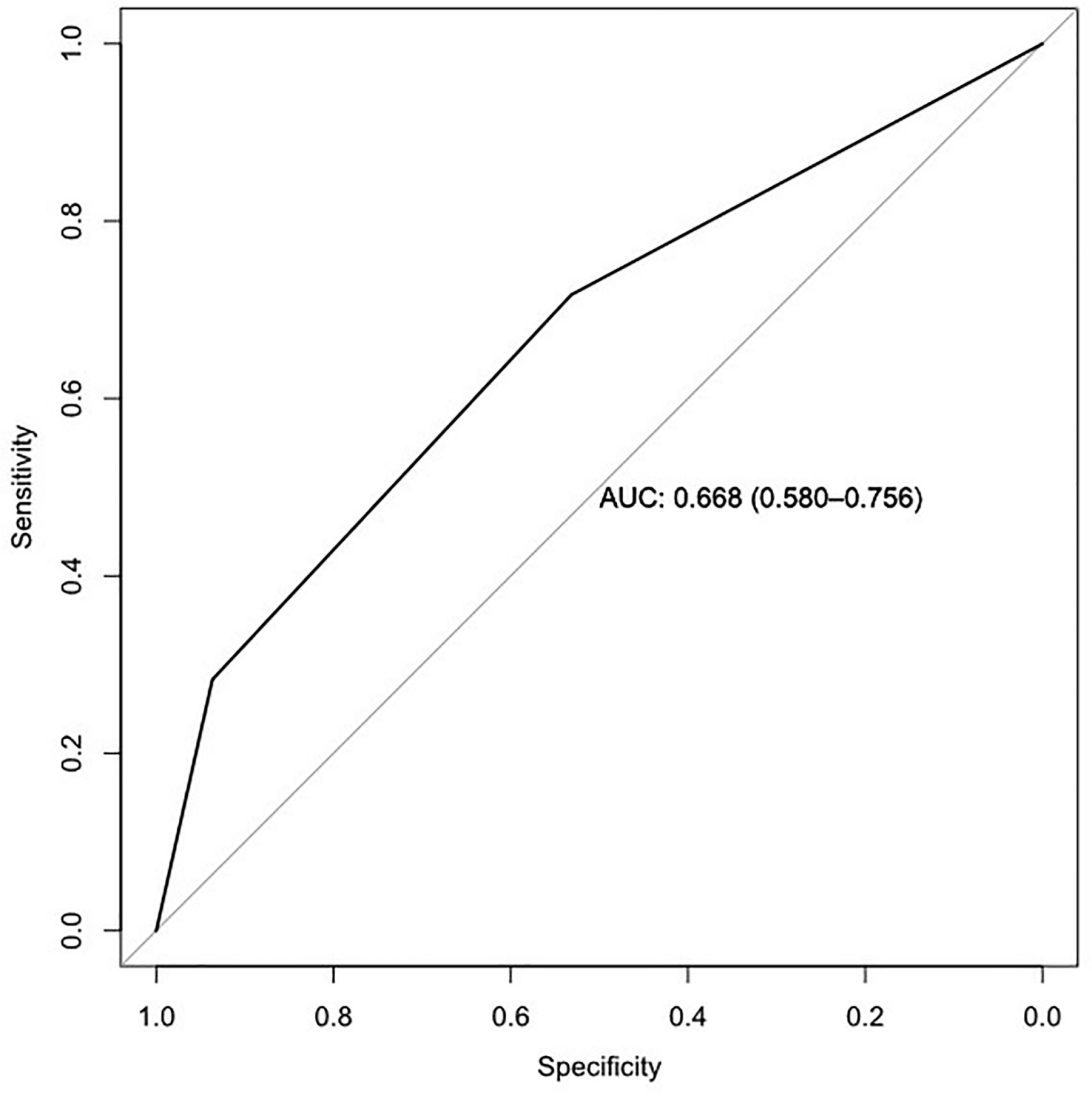
Area Under the Curve from classification and regression tree analysis.

**Fig 3:**
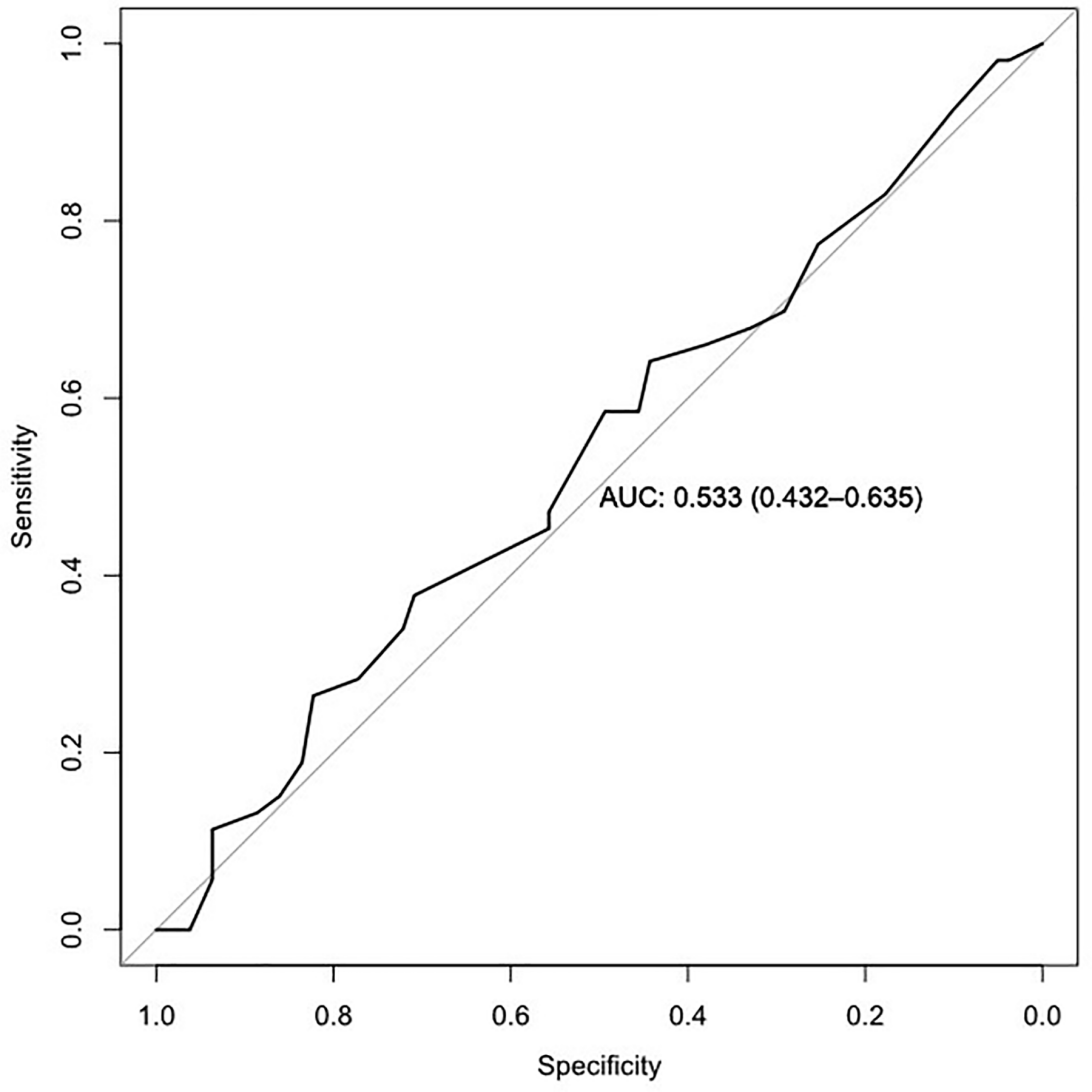
Area Under the Curve from classification and regression tree analysis after 10-fold cross-validation.

The tree from the CART analysis is shown in Figure 1, and the comparison of the true Chikungunya fever status with the predicted status is shown in Table 3. The predictors identified in the tree are pain in the knees and pain in the shoulders. Applying this tree would identify 67.4% of patients in our dataset correctly as either having acute CHIKV infection or not. The majority (71.7%) of the acute CHIKV cases would have been missed. While the corresponding naïve AUC was 67% (95% CI: 58%−76%) (Figure 2), the 10-fold cross-validation resulted in a cross-validated AUC of 53% (95% CI: 43%−63%) (Figure 3).

**Table 3:** True versus predicted CHIKV infection status from classification and regression tree analysis (N = 132).

	True Status	
Predicted Status	Negative	Positive
Negative	74	38
Positive	5	15

**Fig 4:**
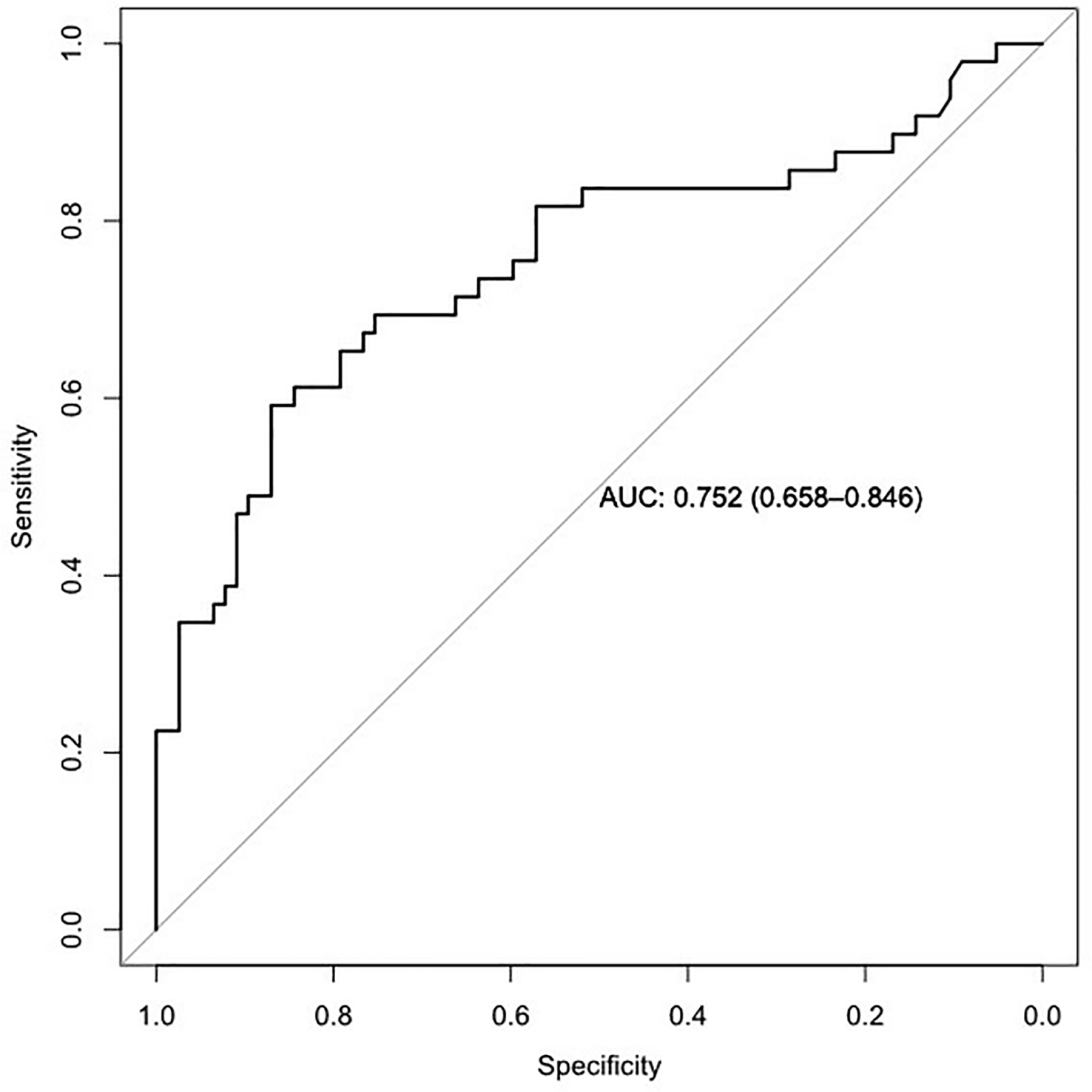
Area Under the Curve from lasso regression analysis.

**Fig 5:**
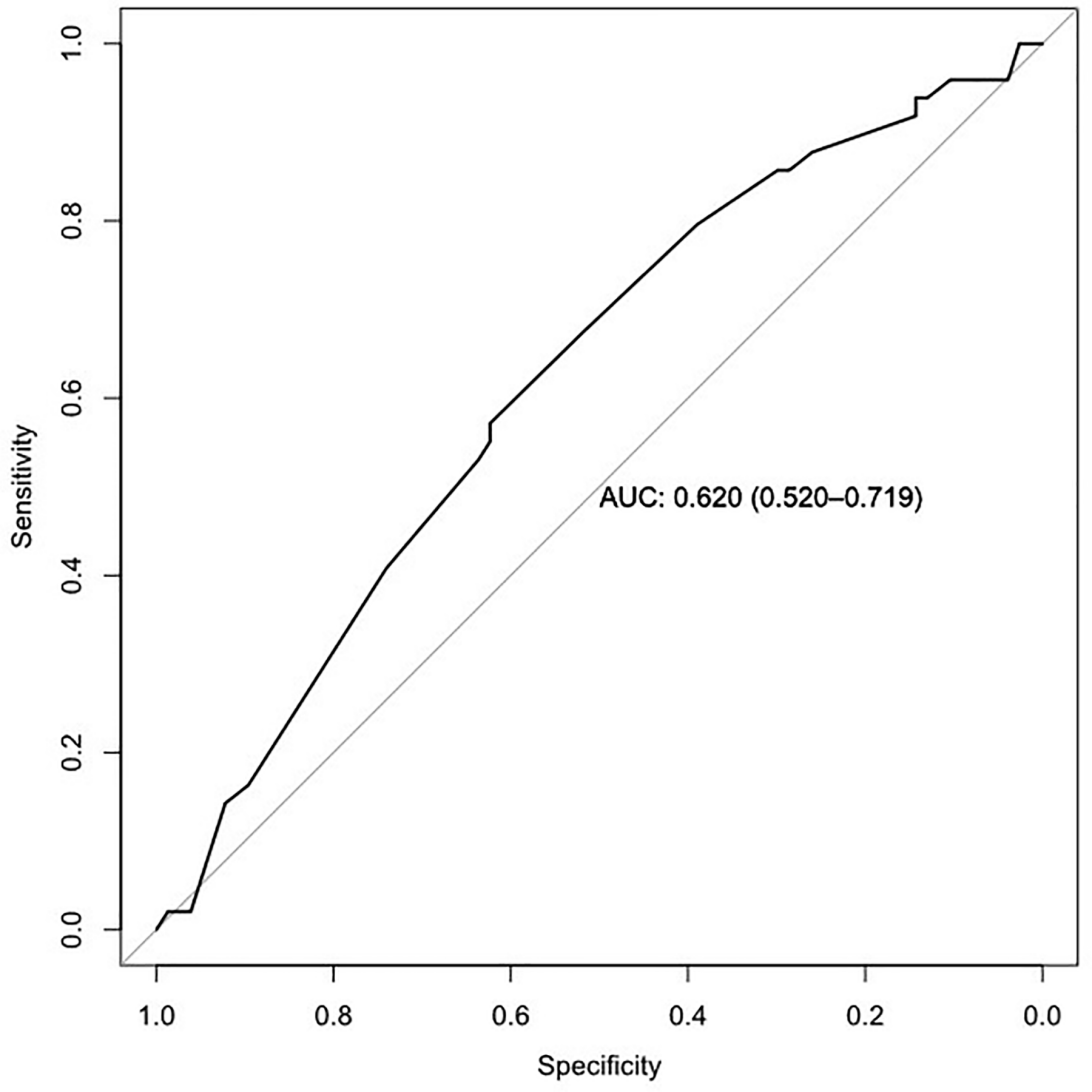
Area Under the Curve from lasso regression analysis after 10-fold cross-validation.

The model resulting from logistic regression analysis with lasso penalty is shown in S3 Box, and the comparison of the true Chikungunya fever status with the predicted status is shown in Table 4. Applying this model resulted in 61.1% correctly classified individuals, and all patients without active CHIKV would have been correctly classified as such. However, 79.6% of the acute CHIKV cases would have been missed. The corresponding naïve area under the ROC curve was 75% (95% CI: 66%−85%) (Figure 4), while the respective cross-validated AUC was 62% (95% CI: 52%−72%) (Figure 5).

**Table 4:** True status versus predicted CHIKV infection status from lasso regression analysis (N = 126).

	True Status	
Predicted Status	Negative	Positive
Negative	77	39
Positive	0	10

Six records were dropped due to missing data.

**Fig 6:**
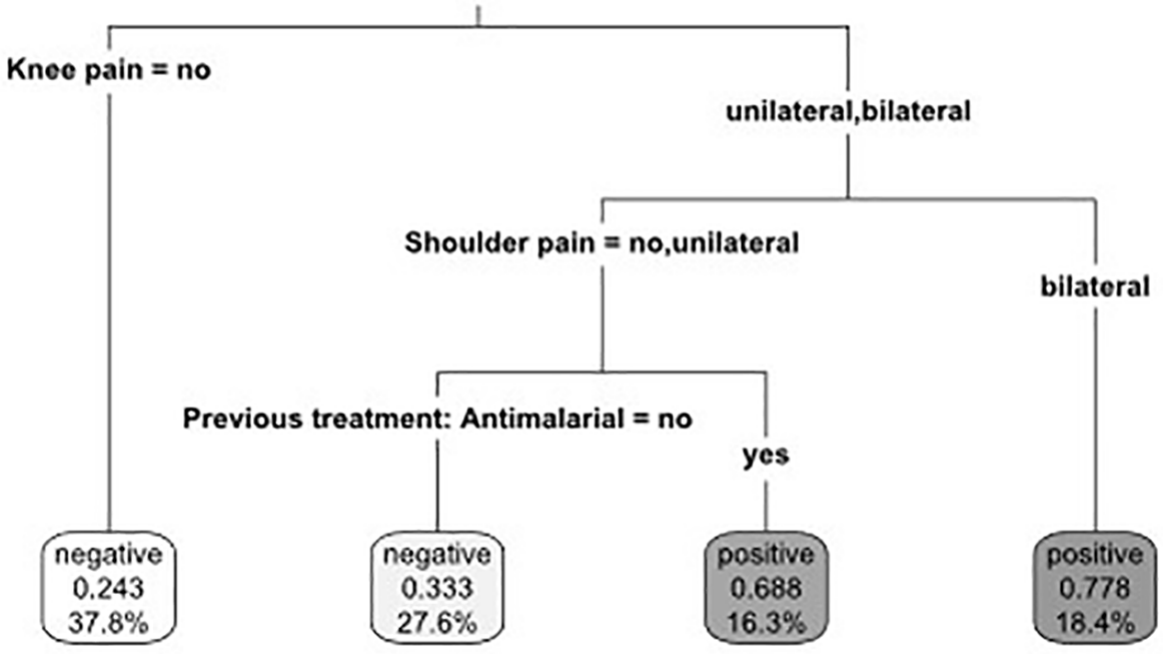
Classification and regression tree analysis after exclusion of 34 patients with acute malaria infection (N = 98).

For the exploratory analysis for chikungunya fever as differential diagnosis to malaria, the 34 participants with pf + pan positive malaria RDT result (see Table 2) were excluded. Among the remaining 98 patients, the respective tree from the CART analysis with cost-complexity parameter chosen by cross-validation is shown in Figure 6, and the comparison of the true Chikungunya status with the predicted status is shown in Table 5. This correctly identified 72.4% of patients, with the performance of the CART analysis being similar compared to the complete case analysis. The lasso logistic regression analysis on this subset with 10-fold cross-validation did not reveal any predictor of CHIKV infection, and with all included subjects being predicted to be negative for chikungunya, the apparent area under the ROC curve was 50%.

**Table 5:** True status versus predicted CHIKV infection status from classification and regression tree analysis after exclusion of 34 malaria cases (N = 98).

	True Status	
Predicted Status	Negative	Positive
Negative	46	18
Positive	9	25

## Discussion

This was the first study attempting to generate an evidence-based clinical case definition for chikungunya fever in a Sub-Saharan African patient population residing in a malaria-endemic region. In summary, we were not able to establish an accurate case definition based on easily recognisable clinical signs and symptoms from the data obtained from this study, which underscore the complexity of diagnosing chikungunya fever in settings where multiple febrile illnesses co-circulate.

The overall prevalence of acute CHIKV infection in our study population was 40.2%, with a substantial proportion (62.3%) of cases detected solely through IgM positivity. This finding highlights the importance of serological assays in diagnosing CHIKV, especially when RT-PCR is not always available. The relatively high percentage of IgM-only positives, combined with the presence of IgG in many of these cases, indicates that a significant portion of CHIKV infections might be missed if only RT-PCR is used for confirmation.

Our CART analysis identified pain in the knees and shoulders as potential predictors of acute CHIKV infection. However, the model only had a moderate, naïve AUC of 67% in identifying CHIKV cases, and only correctly classified 67.4% of patients. The substantial miss rate (71.7% of acute cases) suggests that while these symptoms are indicative, they are not sufficiently discriminatory on their own to reliably diagnose CHIKV infection in a clinical setting. Furthermore, cross-validation revealed an even lower cross-validated AUC of 53%, pointing to the limitations of the CART model and the need for external validation.

The logistic regression model performed slightly better, with a naïve AUC of 75% and correctly classifying 69% of patients. This model showed promise in identifying non-CHIKV cases accurately but still missed a high proportion (79.6%) of acute CHIKV infections. The lower cross-validated AUC of 62% further suggests that while logistic regression may offer some improvements, it is still limited in its practical application without additional contextual or serological information. Our exploratory analysis focusing on patients without malaria co-infection revealed a CART model that performed similarly to the full analysis, identifying 72.4% of CHIKV patients correctly. However, the logistic regression did not yield any predictors, likely due to the limited sample size and variability within the malaria-free subset. This highlights the influence of co-infection on diagnostic accuracy and the necessity of differentiating CHIKV from other febrile illnesses like malaria and dengue, which are highly prevalent in the region.

Notable strengths of our study include broad inclusion criteria, consecutive enrolment, and embedment in routine clinical care during an ongoing outbreak situation, which in combination increases the representativeness of the study population to the target population. Other notable strengths were strong safeguards for data accuracy, e.g., standardized case report forms and double data entry, and high laboratory quality standards at INRB. Main limitations include, first its confinement to a single site, which might have resulted in missing important regional variation in symptomatology and hence may have contributed to the absence of strong diagnostic signals in our analysis. Second, since this study took place in a setting where a multitude of pathogens, among others Dengue virus, co-circulate in the population, there may have been some cross-reactivity with our IgM assay or persistence of IgM-positivity from past chikungunya infection. Third, a rapidly declining outbreak trajectory prevented us from enrolling a higher number of patients into our study, thereby limiting the power of our statistical analysis particularly in the sub-group among malaria-negative patients. Further, being a health centre-based study without community outreach, we might have missed some patients with limited access to health care.

Our findings underline the difficulties in developing a robust clinical case definition for CHIKV in areas with high disease burden and diverse aetiologies of acute febrile illnesses. Instead, simple case definition purely based on easily identifiable signs and symptoms, serological tests, and epidemiological context may be necessary to improve diagnostic accuracy. Further, improved availability and quality of rapid diagnostics for CHIKV, malaria, and dengue coupled with better adherence to their test results for treatment decisions is needed.

## Conclusions

While our study provides valuable insights into the clinical presentation of CHIKV in the DRC and highlights the challenges of diagnostic model development in resource-limited settings, further research is needed to refine and validate a practical case definition for CHIKV. This should ideally involve larger, prospective, multicentric studies that incorporate diverse clinical, geographical, and epidemiological data to enhance the reliability of clinical CHIKV diagnosis in Sub-Saharan Africa.

## Supporting information

S1 Annex Case Report Form. (PDF)

S1 Table Additional clinical characteristics by CHIKV infection status among all included participants (N = 132). (DOCX)

S1 Box Logistic regression model with lasso penalty (chosen by 10-fold cross-validation). (DOCX)

S1 Checklist Questionnaire on inclusivity in global research. (DOCX)
