# Supplementary material for: Developing a clinical case definition for Chikungunya fever in the Democratic Republic of the Congo
Source: PLoS One. 2026 Aug 6;21(8):e0355216. doi: 10.1371/journal.pone.0355216 (PMC13446706; doi:10.1371/journal.pone.0355216)
Supplement: Supplementary file 1 [file pone.0355216.s001.pdf]

[redacted]

FICHE D'INVESTIGATION DES CAS DE FIEVRE OU ARTHRALGIE AIGUE

Date de consultation:___/____/______

A. Depuis combien de jours avez-vous de symptômes ? ____jours

B. Avez-vous fièvre ou arthralgie? ☐ oui ☐ non

Si moins de une semaine à la 1ière question et oui à 2ième question, **la personne est éligible pour l'étude.** Vous pouvez lui présenter l'étude, lui faire signer le consentement éclairé, et compléter le questionnaire. Autrement, la personne n'est pas éligible.

Numéro unique d'identification: ___________________

1. Nom :_________________________ Post-nom :___________________________

   Prénom :____________________________

2. Date de naissance: ___/___/______ Age :

3. Sexe : ☐ M ☐ F

4. Quartier de résidence : ________________Aire de santé :_________________

   Zone de santé :_____________ Numéro de téléphone :___________________

5. Etes-vous enceinte : ☐ oui ☐ non

   Date de dernières règles: __ /__ /______

6. Dans le dernier mois, y-a-t-il eu des cas de fièvre ou arthralgie aigue chez un ou plusieurs membres de votre ménage ? ☐ oui ☐ non ☐ ne sait pas

   Si oui, combien ?________

7. Avez-vous souffert régulièrement des douleurs articulaires dans le passé (avant cette épisode de maladie) ? ☐ oui ☐ non ☐ ne sait pas

   Si oui, quand et pour combien de temps ?_______________________________

   ____

Tableau clinique

8. Symptômes actuels

   Fièvre ☐ oui ☐ non Température : _______°C

   Frissons ☐ oui ☐ non Céphalées ☐ oui ☐ non

   Vertige ☐ oui ☐ non Toux ☐ oui ☐ non

   Rhinite ☐ oui ☐ non Dyspnée ☐ oui ☐ non

   Douleur thoracique ☐ oui ☐ non

[redacted]

Eruption cutanée ☐ oui ☐ non ☐ ne sait pas

Localisation :______________________________

Prurit ☐ oui ☐ non

Anorexie ☐ oui ☐ non Asthénie/fatigue ☐ oui ☐ non

Douleurs abdominales ☐ oui ☐ non

Nausée/Vomissements ☐ oui ☐ non

Diarrhée ☐ oui ☐ non Hémorragie ☐ oui ☐ non

Localisation____

Inconfort/douleur (rétro)oculaire ☐ oui ☐ non

Injection conjonctivale ☐ oui ☐ non

Lombalgie ☐ oui ☐ non Myalgie ☐ oui ☐ non

Arthralgie ☐ oui ☐ non Localisation :

A examiner: cochez avec une croix les articulations avec douleur

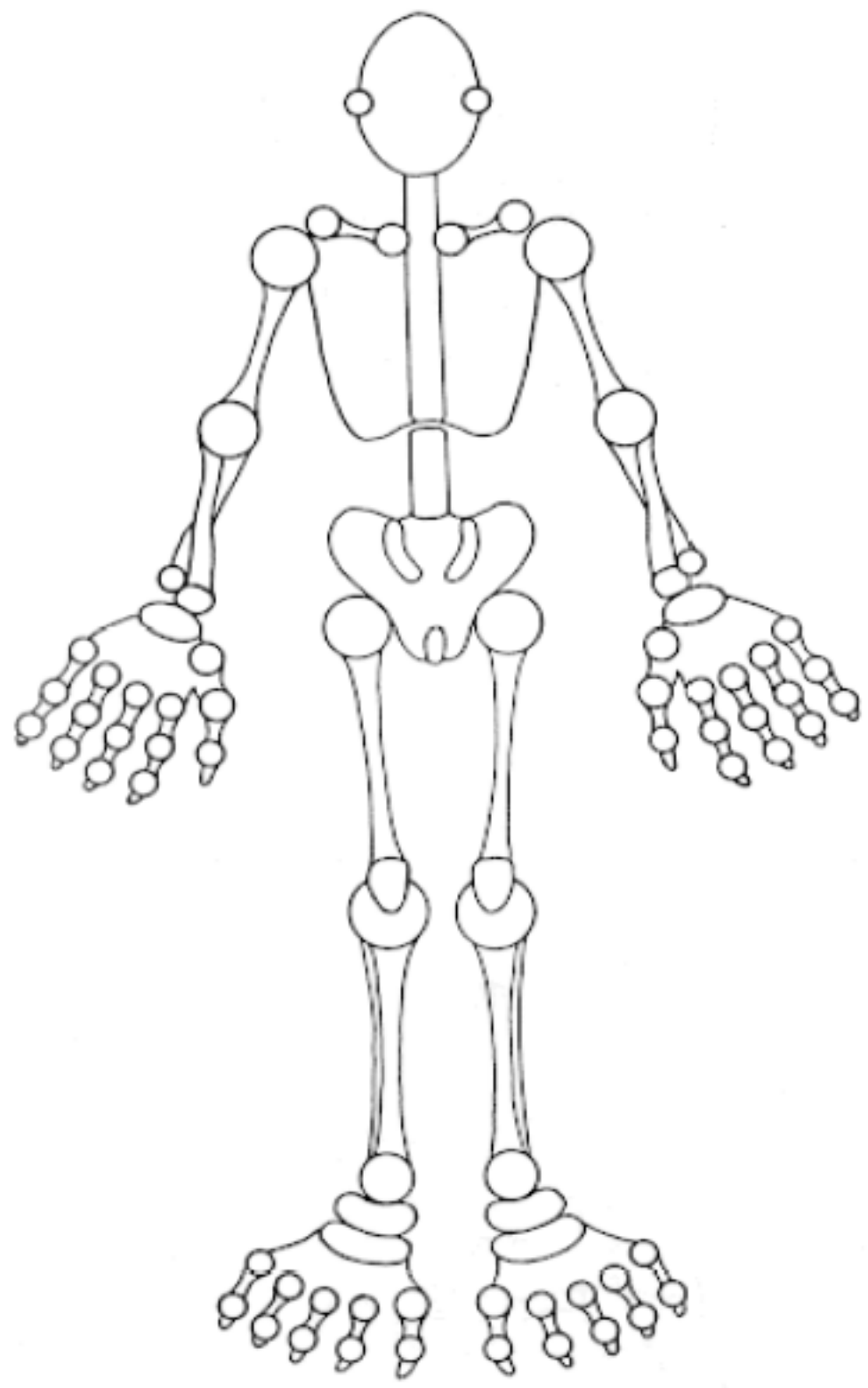

A examiner: cochez avec une croix les articulations enflées

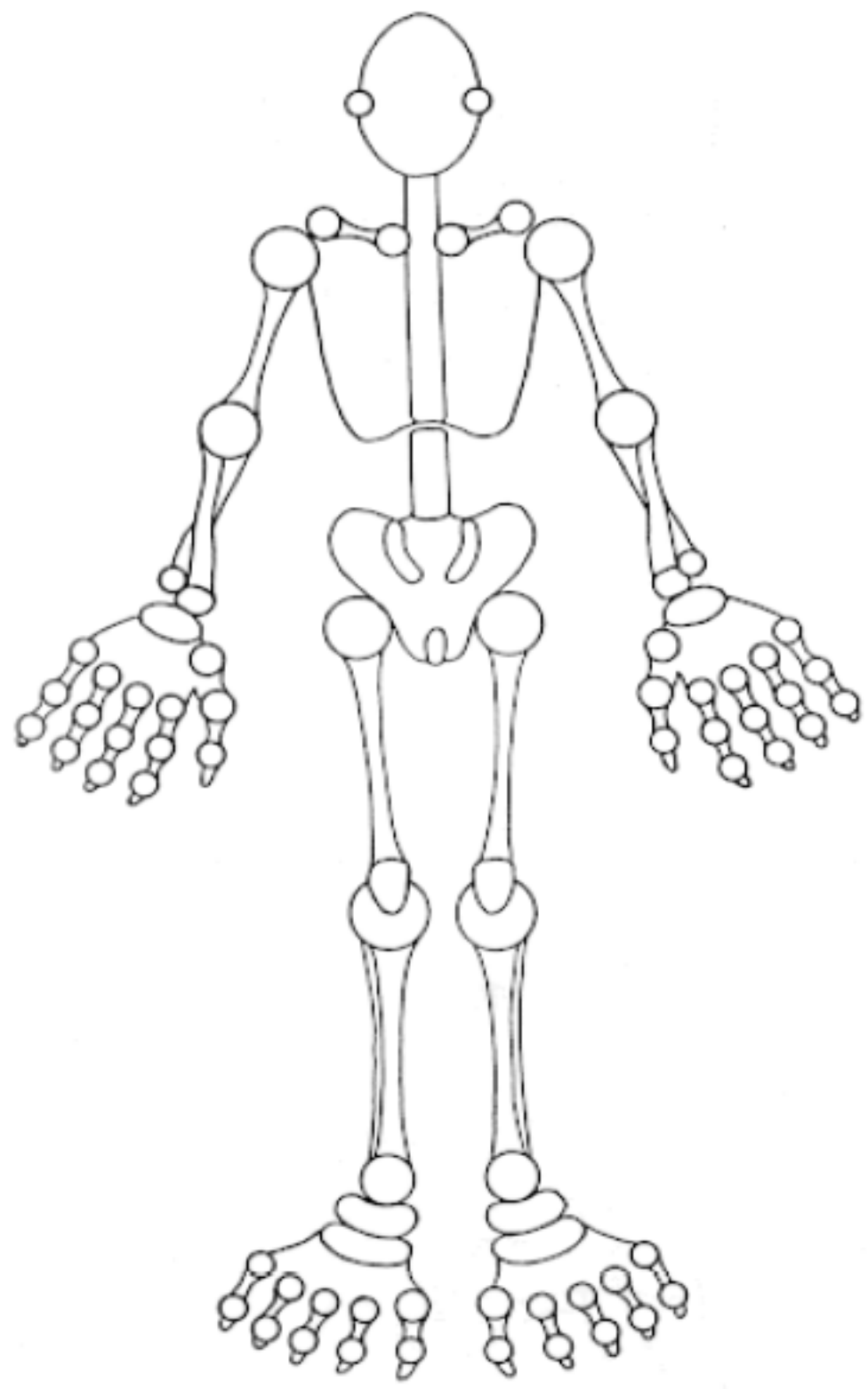

[redacted]

Autres symptômes : ☐ oui ☐ non

Si oui, lesquels:____

9. Avez-vous pris un traitement lors de cet épisode aigu? ☐ oui ☐ non

   Si oui, le(s) quel(s) :

   ☐ antipyrétique ☐ anti-paludisme ☐ antibiotique

   ☐ traditionnel ☐ autre. Si autre, le(s) quel(s) :

   ____

   ____

   ____

Laboratoire

10. Résultat TDR Paludisme : ☐ Pf ☐ Pan ☐ Négatif

    ☐ Pas Disponible ☐ Non-fait

11. Echantillon de sang (tube EDTA) collecté : ☐ oui ☐ non

    Si non, pourquoi ?____

12. Echantillon de sang (papier filtre) collecté : ☐ oui ☐ non

13. Si non, pourquoi ?____

14. Causes probables de la fièvre ou arthralgie aigue:

    ____

    ____

    ____

15. Traitement prescrit :

    ____

    ____

    ____

16. Hospitalisation : ☐ oui ☐ non

Signature de l'enquêteur
