# Supplementary material for: Developing a clinical case definition for Chikungunya fever in the Democratic Republic of the Congo
Source: PLoS One. 2026 Aug 6;21(8):e0355216. doi: 10.1371/journal.pone.0355216 (PMC13446706; doi:10.1371/journal.pone.0355216)
Supplement: Supplementary file 2 [file pone.0355216.s002.docx]

**S2 Table: Additional clinical characteristics by CHIKV infection status among all included participants (N=132).**

|  | **CHIKV infection** | | | |  | |
| --- | --- | --- | --- | --- | --- | --- |
|  | **Negative** | | **Positive** | | **Total** | |
|  | **N=70** | **%** | **N=53** | **%** | **N=132** | **%** |
| **Arthralgia: Neck pain** | |  |  |  |  |  |
| no | 71 | 89.9 | 49 | 92.5 | 120 | 90.9 |
| unilateral | 5 | 6.3 | 4 | 7.5 | 9 | 6.8 |
| bilateral | 3 | 3.8 | 0 | 0.0 | 3 | 2.3 |
| **Arthralgia: Neck swelling** | |  |  |  |  |  |
| no | 79 | 100.0 | 53 | 100.0 | 132 | 100.0 |
| yes | 0 | 0.0 | 0 | 0.0 | 0 | 0.0 |
| **Arthralgia: Shoulder pain** | |  |  |  |  |  |
| no | 64 | 81.0 | 36 | 67.9 | 100 | 75.8 |
| unilateral | 5 | 6.3 | 1 | 1.9 | 6 | 4.5 |
| bilateral | 10 | 12.7 | 16 | 30.2 | 26 | 19.7 |
| **Arthralgia: Shoulder swelling** | |  |  |  |  |  |
| no | 79 | 100.0 | 53 | 100.0 | 132 | 100.0 |
| yes | 0 | 0.0 | 0 | 0.0 | 0 | 0.0 |
| **Arthralgia: Elbow pain** | |  |  |  |  |  |
| no | 51 | 64.6 | 33 | 62.3 | 84 | 63.6 |
| unilateral | 4 | 5.1 | 3 | 5.7 | 7 | 5.3 |
| bilateral | 24 | 30.4 | 17 | 32.1 | 41 | 31.1 |
| **Arthralgia: Elbow swelling** | |  |  |  |  |  |
| no | 79 | 100.0 | 52 | 98.1 | 131 | 99.2 |
| unilateral | 0 | 0.0 | 1 | 1.9 | 1 | 0.8 |
| bilateral | 0 | 0.0 | 0 | 0.0 | 0 | 0.0 |
| **Arthralgia: Wrist pain** | |  |  |  |  |  |
| no | 44 | 55.7 | 23 | 43.4 | 67 | 50.8 |
| unilateral | 1 | 1.3 | 0 | 0.0 | 1 | 0.8 |
| bilateral | 34 | 43.0 | 30 | 56.6 | 64 | 48.5 |
| **Arthralgia: Wrist swelling** | |  |  |  |  |  |
| no | 71 | 89.9 | 47 | 88.7 | 118 | 89.4 |
| unilateral | 0 | 0.0 | 0 | 0.0 | 0 | 0.0 |
| bilateral | 8 | 10.1 | 6 | 11.3 | 14 | 10.6 |
| **Arthralgia: Hand pain** | |  |  |  |  |  |
| no | 57 | 72.2 | 38 | 71.7 | 95 | 72.0 |
| unilateral | 2 | 2.5 | 1 | 1.9 | 3 | 2.3 |
| bilateral | 20 | 25.3 | 14 | 26.4 | 34 | 25.8 |
| **Arthralgia: Hand swelling** | |  |  |  |  |  |
| no | 77 | 97.5 | 50 | 94.3 | 127 | 96.2 |
| unilateral | 0 | 0.0 | 1 | 1.9 | 1 | 0.8 |
| bilateral | 2 | 2.5 | 2 | 3.8 | 4 | 3.0 |
| **Arthralgia: Hip pain** | |  |  |  |  |  |
| no | 68 | 86.1 | 46 | 86.8 | 114 | 86.4 |
| unilateral | 5 | 6.3 | 2 | 3.8 | 7 | 5.3 |
| bilateral | 6 | 7.6 | 5 | 9.4 | 11 | 8.3 |
| **Arthralgia: Hip swelling** | |  |  |  |  |  |
| no | 79 | 100.0 | 52 | 98.1 | 131 | 99.2 |
| unilateral | 0 | 0.0 | 0 | 0.0 | 0 | 0.0 |
| bilateral | 0 | 0.0 | 1 | 1.9 | 1 | 0.8 |
| **Arthralgia: Knee pain** | |  |  |  |  |  |
| no | 42 | 53.2 | 15 | 28.3 | 57 | 43.2 |
| unilateral | 2 | 2.5 | 3 | 5.7 | 5 | 3.8 |
| bilateral | 35 | 44.3 | 35 | 66.0 | 70 | 53.0 |
| **Arthralgia: Knee swelling** | |  |  |  |  |  |
| no | 79 | 100.0 | 50 | 94.3 | 129 | 97.7 |
| unilateral | 0 | 0.0 | 0 | 0.0 | 0 | 0.0 |
| bilateral | 0 | 0.0 | 3 | 5.7 | 3 | 2.3 |
| **Arthralgia: Ankle pain** | |  |  |  |  |  |
| no | 40 | 50.6 | 25 | 47.2 | 65 | 49.2 |
| unilateral | 1 | 1.3 | 1 | 1.9 | 2 | 1.5 |
| bilateral | 38 | 48.1 | 27 | 50.9 | 65 | 49.2 |
| **Arthralgia: Ankle swelling** | |  |  |  |  |  |
| no | 76 | 96.2 | 49 | 92.5 | 125 | 94.7 |
| unilateral | 0 | 0.0 | 1 | 1.9 | 1 | 0.8 |
| bilateral | 3 | 3.8 | 3 | 5.7 | 6 | 4.5 |
| **Arthralgia: Foot pain** | |  |  |  |  |  |
| no | 66 | 83.5 | 43 | 81.1 | 109 | 82.6 |
| bilateral | 13 | 16.5 | 10 | 18.9 | 23 | 17.4 |
| **Arthralgia: Foot swelling** | |  |  |  |  |  |
| no | 78 | 98.7 | 52 | 98.1 | 130 | 98.5 |
| unilateral | 0 | 0.0 | 1 | 1.9 | 1 | 0.8 |
| bilateral | 1 | 1.3 | 0 | 0.0 | 1 | 0.8 |
| **Previous treatment** | |  |  |  |  |  |
| no | 8 | 10.1 | 3 | 5.7 | 11 | 8.3 |
| yes | 71 | 89.9 | 50 | 94.3 | 121 | 91.7 |
| **Previous treatment: Antipyretic** | | |  |  |  |  |
| no | 8 | 10.1 | 8 | 15.1 | 16 | 12.1 |
| yes | 63 | 79.7 | 42 | 79.2 | 105 | 79.5 |
| missing | 8 | 10.1 | 3 | 5.7 | 11 | 8.3 |
| **Previous treatment: Antimalarial** | | |  |  |  |  |
| no | 48 | 60.8 | 28 | 52.8 | 76 | 57.6 |
| yes | 23 | 29.1 | 22 | 41.5 | 45 | 34.1 |
| missing | 8 | 10.1 | 3 | 5.7 | 11 | 8.3 |
| **Previous treatment: Antibiotic** | | |  |  |  |  |
| no | 58 | 73.4 | 37 | 69.8 | 95 | 72.0 |
| yes | 13 | 16.5 | 13 | 24.5 | 26 | 19.7 |
| missing | 8 | 10.1 | 3 | 5.7 | 11 | 8.3 |
| **Previous treatment: Traditional** | | |  |  |  |  |
| no | 70 | 88.6 | 49 | 92.5 | 119 | 90.2 |
| yes | 1 | 1.3 | 1 | 1.9 | 2 | 1.5 |
| missing | 8 | 10.1 | 3 | 5.7 | 11 | 8.3 |
| **Previous treatment: Other** | |  |  |  |  |  |
| no | 58 | 73.4 | 35 | 66.0 | 93 | 70.5 |
| yes | 13 | 16.5 | 15 | 28.3 | 28 | 21.2 |
| missing | 8 | 10.1 | 3 | 5.7 | 11 | 8.3 |
