# Supplementary material for: Developing a clinical case definition for Chikungunya fever in the Democratic Republic of the Congo
Source: PLoS One. 2026 Aug 6;21(8):e0355216. doi: 10.1371/journal.pone.0355216 (PMC13446706; doi:10.1371/journal.pone.0355216)
Supplement: Supplementary file 3 [file pone.0355216.s003.docx]

**S3 Box: Logistic regression model with lasso penalty (chosen by 10-fold cross-validation)***

Log(odds of Chikv) = -0.75-0.0006*age-0.02*past-symptoms+0.03*cephalea-0.30*abdominal_pain+0.26*bleeding-0.33*(neck_pain=”bilateral”)+0.28*(shoulder_pain=”bilateral”)-0.19*(hand_pain=”unilateral”)+0.23*(hip_swelling=”bilateral”)+0.23*(knee_pain=”bilateral”)+1.27*(knee_swelling=”bilateral”)+0.20*previous_antimalarial_treatment+0.23*(malaria rdt=”negative”)+0.04*(pain in at least one joint)

* six records were dropped due to missing data.
